# Insights into the development of *Ixodes scapularis*: a resource for research on a medically important tick species

**DOI:** 10.1186/s13071-015-1185-7

**Published:** 2015-11-14

**Authors:** Katherine M. Kocan, José de la Fuente, Lisa A. Coburn

**Affiliations:** Department of Veterinary Pathobiology, Center for Veterinary Health Sciences, Oklahoma State University, Stillwater, OK 74078 USA; SaBio, Instituto de Investigación en Recursos Cinegéticos (IREC)–Consejo Superior de Investigaciones Científicas (CSIC)–Universidad de Castilla–La Mancha (UCLM)-Junta de Comunidades de Castilla–La Mancha (JCCM), Ronda de Toledo s/n, 13005 Ciudad Real, Spain; Department of Entomology and Plant Pathology, Oklahoma State University, Stillwater, OK 74078 USA

**Keywords:** *Ixodes scapularis*, Ticks, Tick-borne pathogens, Deer tick, Black-legged tick

## Abstract

Ticks (Acari: Ixodida) are arthropod ectoparasites dependent on a bloodmeal from a vertebrate host at each developmental stage for completion of their life cycle. This tick feeding cycle impacts animal health by causing damage to hides, secondary infections, immune reactions and diseases caused by transmission of pathogens. The genus *Ixodes* includes several medically important species that vector diseases, including granulocytic anaplasmosis and Lyme disease. *I. scapularis*, commonly called the black-legged or deer tick, is a medically-important tick species in North America and therefore was the first tick genome to be sequenced, thus serving as an important resource for tick research. This Primer focuses on the normal developmental cycle and laboratory rearing of *I. scapularis*. Definition of normal morphology, along with a consistent source of laboratory-reared *I. scapularis,* are fundamental for all aspects of future research, especially the effects of genetic manipulation and the evaluation of tick vaccine efficacy. Recent research important for the advancement of tick research, namely the development of tick cell culture systems for study of ticks and tick-borne pathogens, RNA interference for genetic manipulation of ticks and discovery of candidate antigens for development of tick vaccines, are briefly presented along with areas to target for future research.

**Electronic supplementary material:**

The online version of this article (doi:10.1186/s13071-015-1185-7) contains supplementary material, which is available to authorized users.

## Why are ticks important?

Ticks (Acari: Ixodidae) are obligate hematophagous arthropods distributed worldwide. As blood sucking ectoparasites, ticks affect humans and animals by causing allergic reactions, damage to hides, decreased animal production, secondary infections, and by transmission of disease-causing pathogens [1–4]. Ticks have few natural enemies and, despite ongoing control efforts, they continue to be a serious threat to human and animal health. Traditional control methods, based on chemical acaricides, have been only partially successful [5, 6], and chemical residues often contaminate the environment and milk and meat products. Importantly, intensive use of acaricides has resulted in the selection of acaricide-resistant ticks [7, 8], a growing problem affecting cattle production worldwide [9–12] and the high cost of developing new acaricides discourages industry production [12]. New control strategies for ticks are therefore needed, and tick vaccines appear to be a promising and sustainable control approach [6, 8, 14–20]. However, development of new and novel vaccines for control of ticks and tick-borne pathogens will require definition of the molecular basis for tick biology and tick-pathogen interactions for discovery of genes/gene products that could be targeted as candidate vaccine antigens [20].

## Why focus research efforts on *I. scapularis*?

Tick and tick-borne disease research is a priority because of the increasing global burden of infectious diseases and the one-health approach for developing control strategies for zoonotic diseases. Notably, *I. scapularis* is a major vector of pathogens in North America that cause diseases in humans and animals, including *Borrelia burgdorferi* (Lyme disease), *Anaplasma phagocytophilum* (animal and human granulocytic anaplasmosis, HGA), *Babesia microti* (rodent and human babesiosis), *Babesia odocoilei* (cervid babesiosis) and Powassan encephalitis virus (PWE) [21]. *I. scapularis,* commonly called the black-legged or deer tick, is a 3-host tick, and the larva, nymph and adult stages feed on separate hosts [22–27]. *I. scapularis* is distributed in North America from southeastern Canada to Saskatchewan, along the Atlantic coast and throughout the Eastern half of the U.S. to eastern Texas, Oklahoma and Florida, and a second species, *I. pacificu*s, is found on the west coast. Other *Ixodes* spp. are common in Europe and other areas of the world. For example, in Europe, *I. ricinus* transmits *A. phagocytophilum*, the etiologic agent of tick-borne fever in sheep and other ruminants, and also the emerging disease of humans, HGA [21, 22]. In the U.S. *I. scapularis* has a two-year life cycle that varies between geographic regions [23–27]. In the northeastern U.S., nymphs are active during late spring and early summer when they are most likely to transmit pathogens to humans [28], while in the southcentral U.S. *I. scapularis* is active in the fall and the immature stages feed predominantly on lizards which are not as likely to serve as reservoir hosts for pathogens [24, 25]. In all regions, adult ticks feed on larger mammals, including deer, livestock, carnivores and humans [23–28]. The 2-year *I. scapularis* life cycle in the northeastern U.S. begins in late summer when larval ticks feed on small mammals and then overwinter and feed as nymphs during the following spring. The adults then feed on large mammals in the fall of the same year [27].

The importance of *I. scapularis* as a vector of pathogens has led to this tick species being a primary focus for research. The selection of *I. scapularis* as the first tick genome to be fully sequenced contributes to this research focus, and the findings from this genomic information and its analysis serve as a model for research on other *Ixodes* spp., most notably *I. ricinus*, the medically important tick counterpart in Europe. Current research on *I. scapularis* includes definition of the genetic basis of tick-pathogen interactions, acaricide resistant genotypes, development of genetic transformation systems, selection of candidate vaccine antigens and development of tick vaccines [20].

**Fig. 1:**
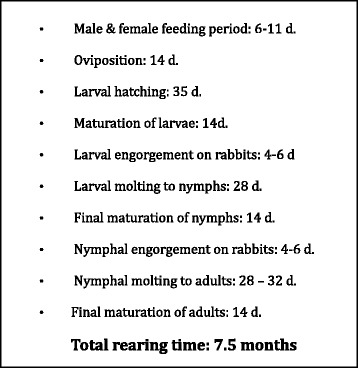
Time sequence for rearing *I. scapularis* in the laboratory

Laboratory-reared *I. scapularis* are essential for research in order to provide a source of uniform, pathogen free ticks. Rickettsial pathogens that infect *I. scapularis* are transmitted from stage to stage (transstadial transmission) but not by transovarial transmission via eggs. Therefore, subsequent generations of laboratory reared ticks will be pathogen free. While *I. scapularis* is considerably more difficult to rear, the life cycle can be completed faster in the laboratory (7.5 months as opposed to two years in nature, Fig. 1). The Centralized Tick Rearing Facility, Department of Entomology and Plant Pathology, Oklahoma State University, have devised methods for large-scale production of *I. scapularis*.

Knowledge of the normal development cycle of *I. scapularis* is essential in order to fully assess the effects of experimental and genetic tick manipulations. For this reason, we documented the normal developmental cycle of *I. scapularis* from mating, oviposition and egg hatching, through the feeding, engorgement and molting of each life stage.

## Developmental cycle of *I. scapularis*

Morphologic details of the *I. scapularis* developmental stages are presented in the Additional files 1 and 2 in both a poster and video format.

### Mating and engorgement

While many species of male ixodid ticks feed intermittently on the host preceding mating, a bloodmeal is not a prerequisite for *I. scapularis* mating, and mating can occur off host. Males copulate multiple times with the same or different females, and often stay attached to the female ticks throughout the 6–11 day feeding period. During mating, the male tick inserts the hypostome and chelicerae into the female’s genital opening for transfer of the spermatophore, while the palps are splayed to the sides. Successful mating is required for the onset of the rapid stage of engorgement, after which the female drops from the host. In the absence of males, unmated females remain on host and feed slowly for longer periods [23].

### Oviposition and emergence of larval ticks

After female ticks complete mating and the rapid stage of engorgement, they drop off the host. Oviposition then commences and is completed within 14 days. Multicellular eggs are expelled from the genital pore on the ventral side of the female and are passed over the capitulum where they are coated with wax extruded from two porous areas on the base of the capitulum. The wax protects the eggs from drying and also loosely binds the eggs together to form an egg mass. Within 35 days the eggs embryonate and prior to hatching the larval body and legs can be seen through the transparent shell. Hatching occurs rapidly as the egg shell ruptures along a suture line. The legs and mouthparts of the newly-hatched larvae are initially transparent, but after 14 days of maturation become sclerotinized. The larvae then quest together in groups for hosts.

### Feeding, molting and emergence of nymphs and adults

Larvae feed 4 days after which they engorge, drop off host and then molt in approximately 28 days to the nymphal stage. The exoskeleton opens on a rupture line at the base of the capitulum. The legs are the last to detach from the exoskeleton. The legs and mouthparts of the newly-molted nymphs are transparent but darken during the 14 day maturation period as sclerotin forms and causes stiffening of the cuticle. After this period, the nymphs quest, attach and feed on the host. Nymphs feed for 4–6 days, after which they drop off the host and molt to the adult (male or female) stage, a process that requires 4–5 weeks. After a maturation time of 14 days, the cuticle stiffens with the formation of sclerotin and the males are able to mate with females either off host or during the feeding cycle on large mammals.

### Current advances and future research

General advances on ticks and tick-borne pathogens and targeted areas for future research are presented because of their implications for ixodid tick species.

## Ticks and tick-borne diseases -Three advances made in the last decade

### Development of tick cell cultures for study of ticks and tick-borne pathogens

Establishment of continuous tick cell lines was first reported by Varma et al. [31] and subsequently over 40 cell lines are now reported including ones from several tick species [32–35]. Development of these tick cell lines has been an important breakthrough because they have provided a venue for in vitro studies on tick biology and tick-pathogen interactions and also have reduced the dependence on animals for research on ticks and tick-borne pathogens. Cell lines derived from *I. scapulari*s were the first to be used for propagation of several important tick-borne pathogens, including *Anaplasma*, *Borrelia, Ehrlichia, Rickettsia,* and many viruses [34]. Interestingly, *Ixodes*-derived cell lines were found to support the growth of pathogens for which this tick is not the natural vector, such as *A. marginale* [32, 35]. Tick cell culture has been recently applied to gene silencing and genetic transformation studies, and for characterization of tick-pathogen interactions using omics technologies [20, 34–37].

### RNA interference for genetic manipulation of ticks and analysis of the impact gene expression on tick biology and tick-host-pathogen interactions

Tick gene silencing by RNA interference (RNAi), first demonstrated by Aljamali et al. [38], is currently the only means of genetic manipulation of ticks. RNAi has been adapted for use in ticks and tick cell culture [39–41], and has become a valuable tool for functional analyses of tick genes, characterization of the tick-pathogen and tick-host interface and for screening for tick protective antigens [20, 41, 43]. RNAi used in combination with transcriptomics and proteomics has also allowed for identification of genes differentially regulated in ticks in response infection with pathogens [36, 39].

### Discovery of candidate antigens for development of vaccines against ticks and tick-borne pathogens

Ticks vaccines, thus far developed for cattle, have been identified as an important component of future control strategies for both ticks and tick-borne pathogens [20]. The tick-protective antigen, BM86, was first used to develop and market the first cattle vaccine for control of *Rhipicephalus* spp., thus demonstrating the utility of tick vaccines [15–20]. Fundamental toward further development of tick vaccines is the discovery of candidate vaccine antigens [19, 20]. While new candidate antigens are being tested in cattle [20], the continued search for vaccine antigens has been augmented by the availability of genomic sequence information. The genome of *I. scapularis* was the first tick genome to be sequenced but will soon be followed by genomes of other important tick species, including that of *Rhipicephalus microplus* [42], contributing to the discovery of many promising antigens [20, 42, 43]. For example, Subolesin, discovered by expression library immunization and then characterized by RNAi [41, 44] was found to be the ortholog of insect and vertebrate Akirin [45, 46], a transcription factor required for NF-kB-dependent gene expression and regulation of the innate immune response to pathogen infection [37]. The silencing of Subolesin by RNAi resulted in reduced female weight gains, rendered males sterile, and the failure of females to complete mating and feeding reduced or blocked oviposition [46–48] and also interfered with pathogen infection, development and transmission [49, 50]. Molecular interactions between ticks and pathogens are being defined and will increase the range of candidate vaccine antigens that impact both tick biology and tick pathogen infection and transmission, thus providing the opportunity for development of ‘dual target’ vaccines that target ticks and tick-borne pathogens [20, 51–59].

## Ticks and tick-borne diseases -Three areas ripe for research

### Analyses of genome sequence and omics data bases and a systems biology approach for discovery of candidate vaccine antigens

Future vaccines will be dependent on inclusion of key molecules important for tick biology and protective mechanisms. A systems biology approach using the large data bases generated from genomic, proteomic, transcriptomic and metabolomic analyses provides the opportunity to comprehensively define the molecular biology of the tick-host cell interface [20, 42, 60]. These data can then be a resource for discovery of a new and expanded generation of biomarkers and candidate vaccine antigens [35]. In addition, when sequences of multiple tick genomes become available, comparative studies across tick species can be conducted toward development of both species-specific vaccines and those cross-protective among multiple tick species. However, while these data bases are presently becoming a valuable resource, limitations in genome sequence information, assembly and annotation provide challenges for future research involving the comprehensive characterization of the molecular events at the tick-pathogen interface [20]. Design of experiments combining tick transcriptomics and proteomics will be dependent on integration of these large datasets for assessing global transcriptome and proteome changes of specific pathways, such as immune response and apoptosis required for pathogen infection and transmission by ticks [49–51].

## Development of dual target vaccines for control of ticks and tick-borne pathogens

Recent results have clearly demonstrated molecular interactions between ticks and the pathogens that they transmit. Candidate tick antigens have been identified that reduce pathogen infection and transmission while also affecting tick infestations [49–59, 61–64]. Therefore, the development of dual target vaccines that reduce both tick infestations and pathogen infection and transmission appears to be an achievable goal, and the combination of tick- and pathogen-derived antigens should result in development of vaccines for ticks and tick-borne diseases [5, 8, 20, 55].

## Characterization of tick microbiomes

Descriptive characteristics of the tick microbiome, which is the collection of commensal, symbiotic and pathogenic microorganisms that occupy each tick species, were recognized years ago but the ability to fully define and characterize these communities is becoming possible because of rapidly-evolving molecular technologies [65]. The developmental cycles of pathogens are complex and pathogens acquired via the blood meal first must infect gut cells and eventually colonize other tissues, some of which are important for transmission during feeding by subsequent stages. Ticks are also infected with endosymbionts which likely impact tick biology and pathogen infections. The understanding of tick microbiomes and their impact on tick survival and vector competency will enhance the search for candidate vaccine antigens within and among tick species and broadly across arthropod groups [65].

## Conclusions

The genus *Ixodes* includes several species of ticks that are medically important worldwide. Their populations and the pathogens they transmit are expanding posing increased threat to human and animal health. *I. scapularis* is one of the most medically important ticks in the U.S. and has been the first tick genome to be sequenced, providing an important resource for tick and tick-borne pathogen research. Fundamental for future research is a source of laboratory-reared ticks and an understanding of this tick’s normal developmental cycle. In this Primer we detailed the *I. scapularis* developmental cycle, recent advances toward the understanding of *I. scapularis* biology, its role as a vector of pathogens and vaccines development for control of ticks and tick-borne pathogens and areas to target for future research. As part of integrated control programs, tick vaccines promise to be an effective intervention that will reduce the use of acaricides and the selection of acaricide resistant ticks. Because tick species parasitize several vertebrate hosts and share habitat and hosts, development of vaccines cross protective against multiple tick stages, hosts and pathogens should be possible using genome screening and omics technologies to target relevant biological processes for discovery of novel candidate vaccine antigens.

### Ethics

Not application.

## Additional files

Additional file 1:
**Poster of** ***Ixodes scapularis*** **development.** (PDF 2003 kb) Additional file 2:
**Video clip of the development of** ***Ixodes scapularis.*** (MP4 36664 kb)

## Abbreviations

HGA: human granulocytic anaplasmosis
PWE: Powassan encephalitis
RNAi: RNA interference
