# Supplementary material for: Insights into the development of Ixodes scapularis: a resource for research on a medically important tick species
Source: Parasit Vectors. 2015 Nov 14;8:592. doi: 10.1186/s13071-015-1185-7 (PMC4650338; doi:10.1186/s13071-015-1185-7)
Supplement: Additional file 1: — Poster of Ixodes scapularis development. (PDF 2003 kb) [file 13071_2015_1185_MOESM1_ESM.pdf]

Primers on Parasites & Vectors

Insights into the Development of *Ixodes scapularis*: A Resource for Research on a Medically Important Tick Species

Katherine M. Kocan, José de la Fuente & Lisa Coburn

Parasites & Vectors

Introduction, Mating & Female Engorgement

Introduction: *Ixodes scapularis*, the black-legged or deer tick, is a 3-host tick in which each life stage (larva, nymph & adult) feeds on a separate host. This tick transmits pathogens that cause diseases in animals and humans, including *Borrelia burgdorferi* (Lyme borreliosis), *Anaplasma phagocytophilum* (animal and human granulocytic anaplasmosis, HGA), *Babesia microti* (rodent and human babesiosis), *Babesia odocoilei* (cervid babesiosis) and Powassan encephalitis virus (PWE).[1-3] The *I. scapularis* life cycle, which requires 2 years in nature,[4] can be completed in the research laboratory in approximately 7.5 months. The larval stage acquires infection from small animals and the nymphal stage most often transmits the pathogens.

Mating & Engorgement: Mating occurs off of the host and the males do not require a prior bloodmeal. During mating, males insert only the hypostome and chelicerae into the female's genital opening and transfer spermatophores. Males may remain attached to the female throughout the 6-11 day feeding period. Successful mating is required for the onset of the female's rapid stage of engorgement, repletion and ovipostion.

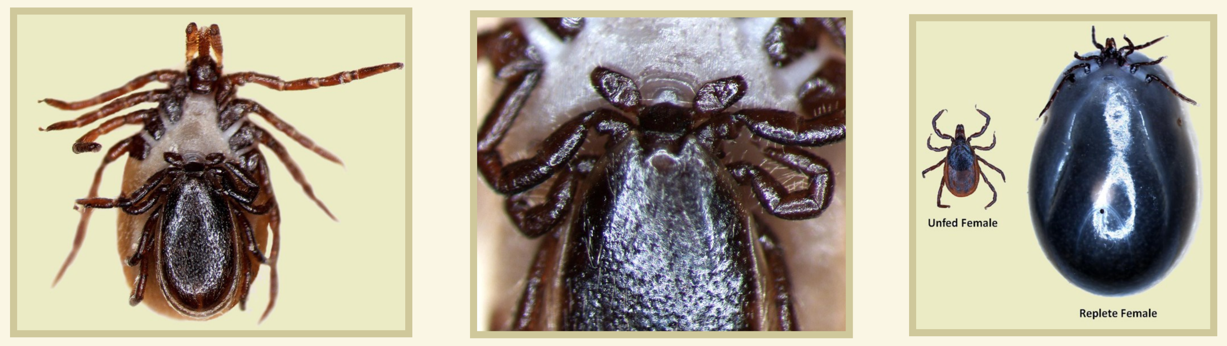

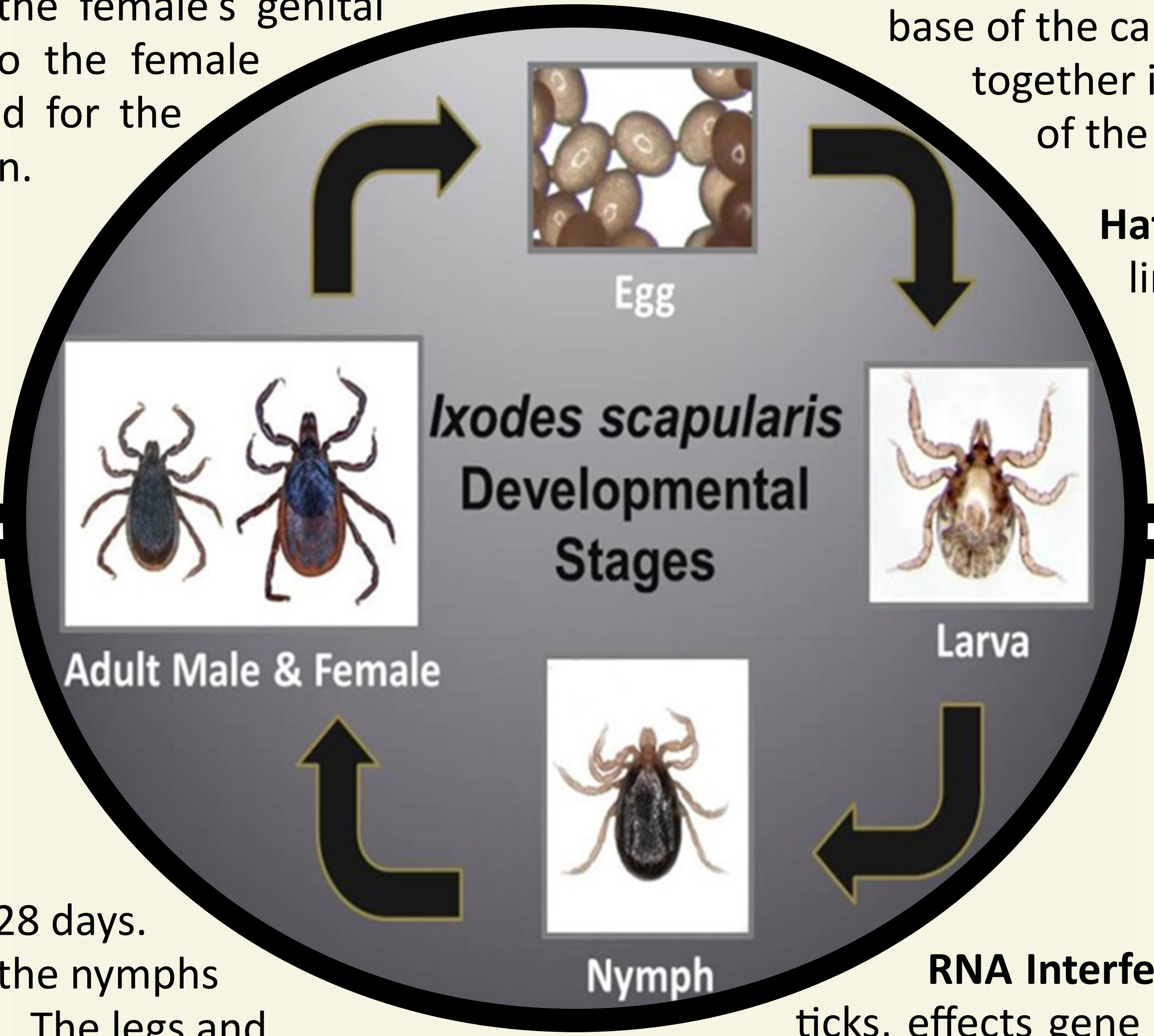

Oviposition and Egg Hatching

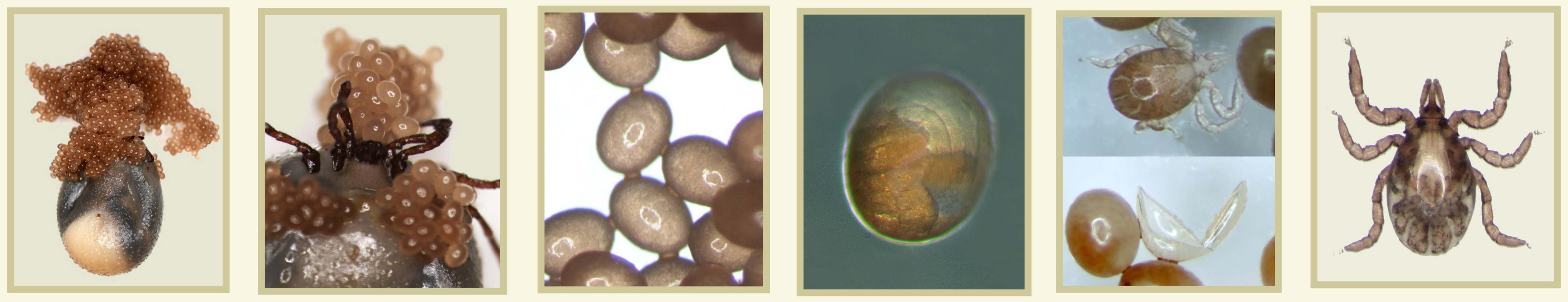

Oviposition: Replete females drop off the host and within 14 days oviposit multicellular eggs which are expelled from the genital opening on the ventral side of the engorged female. The eggs are then passed over the capitulum where they are coated with wax extruded from two porose areas at the base of the capitulum. The wax protects the eggs from drying and also loosely binds them together into an egg mass. Within 35 days the eggs embryonate, and the body & legs of the developing nymphs can be seen through the thin egg shell.

Hatching: The larvae rapidly emerge as the egg shell ruptures along a median line. The legs and mouthparts of the newly-hatched larvae are initially transparent but become sclerotinized as the larvae mature over 14 days. The larvae then seek small mammal hosts, to which they attach, feed for 6 days, and then drop off to molt in the leaf litter.

Nymphal & Adult Engorgement & Molting

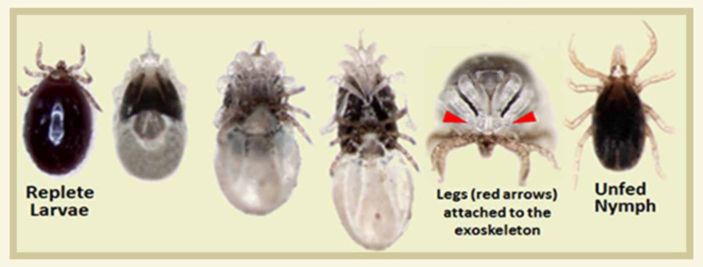

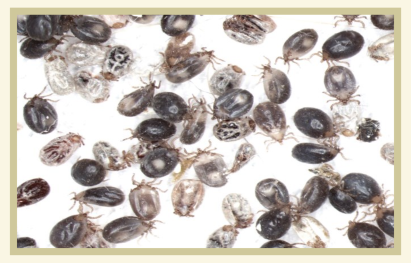

Nymphs molt (ecdysis) and emerge from replete larvae in approximately 28 days. The exoskeleton opens at a rupture line at the base of the capitulum. As the nymphs emerge, the legs (red arrows) are the last to detach from the exoskeleton. The legs and mouthparts of the newly molted nymph are transparent but darken as sclerotization occurs during the 14-day maturation period. Many replete nymphs fail to complete molting and die.

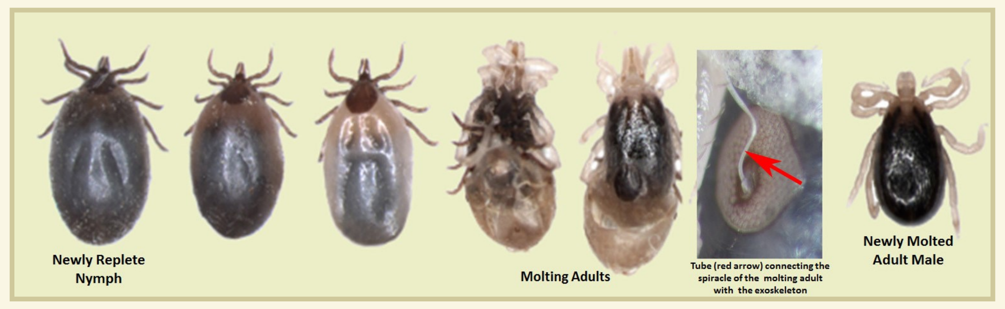

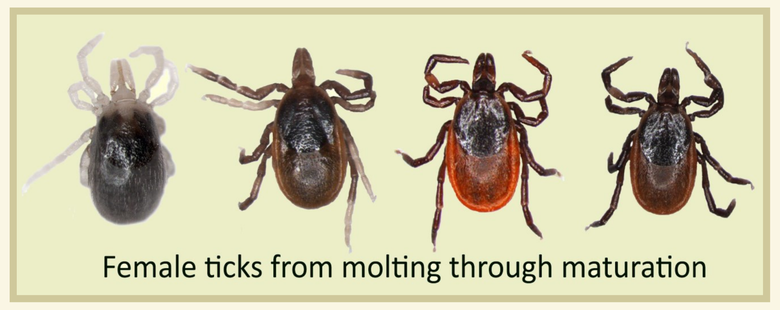

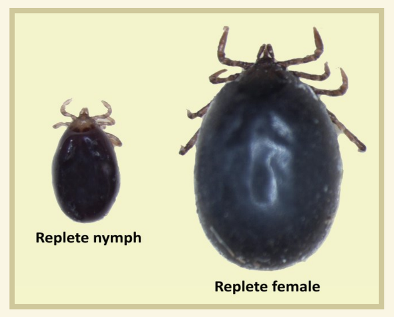

Replete nymphs feed for 4-6 days, drop off the host, and within 4-5 weeks molt to the adult (male or female) stage. Molting is a slow process during which the emerging adults breath via tubes (red arrow) that connect the spiracles of the emerging adult with the exoskeleton. After 14 days, the ticks are able to mate, and attach to and feed on large mammals, thus completing the life cycle.

Impact of Gene Silencing & Pathogen Infection

Injection of ticks with dsRNA

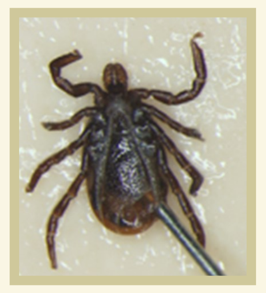

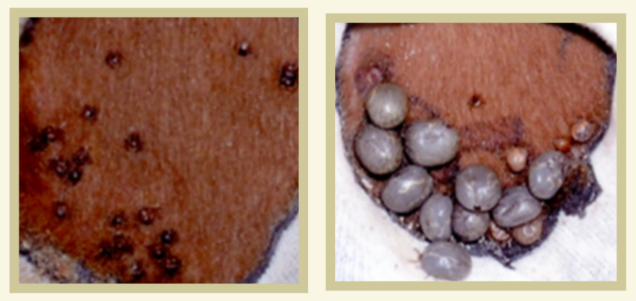

Feeding impaired in injected vs control ticks

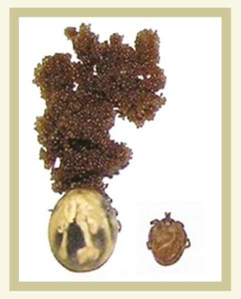

dsRNA-injected ticks had reduced oviposition

RNA Interference (RNAi): RNAi, currently the only method for genetic manipulation of ticks, effects gene silencing after gene-specific double-stranded RNA is injected into the tick. RNAi has become the most valuable tool for functional analyses of ticks, the study of tick gene function, characterization of the tick-pathogen interface and for screening and characterization of tick protective antigens.[5-7] Evidence of gene silencing includes reduced tick weights, failure to mate, failure of females to complete feeding and ovipositon and by reduced egg mass weights.

Tick-Pathogen Interactions: A systems biology approach, combining "omics" technologies, such as transcriptomics & proteomics, was recently used to characterize global tissue-specific responses and signaling pathways of *I. scapularis* in response to infection with *A. phagocytophilum.* The complexity of the tissue responses to infection revealed a dynamic tick-pathogen interface that, when fully defined, will allow for development of novel control strategies for infection and pathogen transmission.

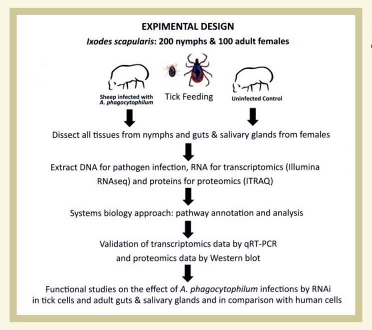

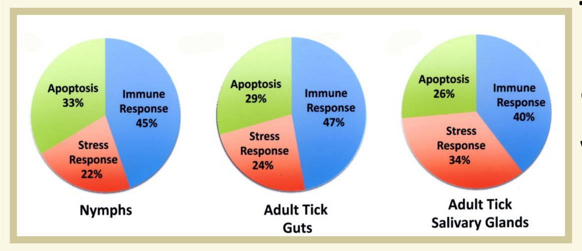

Sources & Further Reading:

(1) Keirans JE, Hutcheson HJ, Durden LA, Klompen JSH. *Ixodes* (*Ixodes*) *scapularis* (Acari: Ixodidae): redescription of all active stages, distribution, hosts, geographical variation, and medical and veterinary importance. J Med Entomol. 1996;33:297–318.

(2) Dennis DT, Nekomoto TS, Victor JC, Paul WS, Piesman J. Reported distribution of *Ixodes scapularis* and *Ixodes pacificus* (Acari: Ixodidae) in the United States. J Med Ento Mol.1 998;35:629–638

(3) Krakowetz CN, Chilton NB. 2015. Sequence and secondary structure of the mitochondrial 16S ribosomal RNA gene of *Ixodes scapularis.* Mol Cell Probes , 2015;29: 35-38

(4) Oliver JH Jr. Lyme borreliosis in the southern United States: a review. J Parasitol 1996;82:926-35.

(5) de la Fuente J, Kocan KM, Almazán C, Blouin EF. RNA interference for the study and genetic manipulation of ticks. Trends Parasitol. 2007;23:427–433.

(6) Aljamali, MN, Bior AD, Sauer JR,, Essenberg RC. RNA interference in ticks: a study using histamine binding protein dsRNA in the female tick *Amblyomma americanum*. Insect Mol Biol. 2003;2:99-305

(7) Kocan KM, Blouin E, de la Fuente J. RNA interference in ticks. JoVE 2011;47: e2474. http://www.jove. com/details.stp?id=2474.

(8) Ayllón N, Villar M, Galindo RC, Kocan KM, Šíma R, López JA, Vázquez J, Alberdi P, Cabezas-Cruz A, Kopáček P, de la Fuente J. Systems biology of tissue-specific response to *Anaplasma phagocytophilum* reveals differentiated apoptosis in the tick vector *Ixodes scapularis.* PLOS Genetics. 2015; 11: e1005120
